# Low reproductive skew despite high male-biased operational sex ratio in a glass frog with paternal care

**DOI:** 10.1186/s12862-015-0469-z

**Published:** 2015-09-03

**Authors:** Alexandra Mangold, Katharina Trenkwalder, Max Ringler, Walter Hödl, Eva Ringler

**Affiliations:** Department of Integrative Zoology, University of Vienna, Althanstrasse 14, A-1090 Vienna, Austria; Messerli Research Institute, University of Veterinary Medicine Vienna, Veterinärplatz 1, A- 1210 Vienna, Austria

## Abstract

**Background:**

Reproductive skew, the uneven distribution of reproductive success among individuals, is a common feature of many animal populations. Several scenarios have been proposed to favour either high or low levels of reproductive skew. Particularly a male-biased operational sex ratio and the asynchronous arrival of females is expected to cause high variation in reproductive success among males. Recently it has been suggested that the type of benefits provided by males (fixed vs. dilutable) could also strongly impact individual mating patterns, and thereby affecting reproductive skew. We tested this hypothesis in *Hyalinobatrachium valerioi*, a Neotropical glass frog with prolonged breeding and paternal care.

**Results:**

We monitored and genetically sampled a natural population in southwestern Costa Rica during the breeding season in 2012 and performed parentage analysis of adult frogs and tadpoles to investigate individual mating frequencies, possible mating preferences, and estimate reproductive skew in males and females. We identified a polygamous mating system, where high proportions of males (69 %) and females (94 %) reproduced successfully. The variance in male mating success could largely be attributed to differences in time spent calling at the reproductive site, but not to body size or relatedness. Female *H. valerioi* were not choosy and mated indiscriminately with available males.

**Conclusions:**

Our findings support the hypothesis that dilutable male benefits - such as parental care - can favour female polyandry and maintain low levels of reproductive skew among males within a population, even in the presence of direct male-male competition and a highly male-biased operational sex ratio. We hypothesize that low male reproductive skew might be a general characteristic in prolonged breeders with paternal care.

**Electronic supplementary material:**

The online version of this article (doi:10.1186/s12862-015-0469-z) contains supplementary material, which is available to authorized users.

## Background

A wide range of mating systems has evolved in sexually reproducing animals [1, 2]. Mating systems are shaped by the two components of sexual selection: direct competition among individuals for access to mates (intrasexual selection) and the actual mating decision (intersexual selection). These two processes act in the context of general traits of a species and the current environmental conditions, and are shaped by specific characteristics of males and females in a certain population [1, 3]. The prevailing mating system in a group of breeding individuals thus dynamically derives from the optimal, or at least available, mating strategies of all the involved individuals at a given time [4, 5].

Reproductive skew, the uneven distribution of reproductive success among individuals, is a common feature of many animal populations [6]. High male reproductive skew occurs if most females show a preference for a single male (or a few males) in the population, or, in turn, if single males can monopolize the majority of receptive females. Several scenarios have been proposed that would predict high or low levels of reproductive skew, respectively. For example, a highly male-biased operational sex ratio (OSR), which will increase the intensity of male competition for females, is expected to increase the variance in reproductive success among males [1, 7]. The OSR of a given population is, in turn, influenced by parameters such as the population-wide sex ratio, potential reproductive rates in males and females, their mating frequencies, and the asynchronous arrival of females in the breeding population [1, 3, 8].

Amphibian mating patterns are typically divided into two broad categories: explosive breeders, with individuals breeding over a few days to a few weeks, and prolonged breeders, with individuals breeding longer than a month. However, these categories should rather be seen as the two ends of a continuum [9]. High mating pressure on females, the short time-frame available for reproduction, scramble mating with associated sperm competition when females get amplexed by multiple males simultaneously, and stray sperm in aquatic environments presumably preclude the possibility of active female choice in explosively breeding species [10; but see 11]. In species with prolonged breeding, females might be able to selectively choose among available males due to the relaxed temporal constraints. Indeed, discriminatory female choice for certain male traits that indicate physical or genetic quality has been found in field studies of various prolonged breeders (e.g. *Engystomops* (*Physalaemus*) *pustulosus*, [12]; *Uperoleia rugosa*, [13]; *Scinax ruber* (*Ololygon rubra*), [14]; *Dendropsophus*, (*Hyla elegans*), [15]). Several studies have also shown female preferences for certain call characteristics in two-speaker phonotaxis experiments in the laboratory, although under more realistic conditions these preferences sometimes diminished or vanished altogether (reviewed in [16, 17]).

Extended breeding periods are expected to lead to high reproductive skew in males [9]. The rationale behind this hypothesis is that the longer time-frame for mating, and the associated asynchronous arrival of females at the reproductive site, theoretically enables single males to monopolize large shares of the total number of approaching females, compared to explosive breeders. In stark contrast to these theoretical predictions, high male mating skew has been reported for many explosively breeding anuran species (cf. [18, 19]), while comparatively low reproductive skew has recently been reported for a prolonged breeding poison frog [20].

A complementary conceptual framework on this issue has been proposed in a recent study [21], suggesting that higher mating skew can be expected in situations where males provide fixed benefits for females (i.e. benefits are not shared or divided between other females under male polygyny, for example ‘good genes’) compared to situations where the provided benefits are dilutable (i.e. benefits are shared between females mating with the same male). One prominent and common example for such a dilutable benefit is male parental performance, as quality and quantity of paternal care can be expected to decrease with an increasing number of eggs/clutches. In amphibians more eggs/clutches are usually associated with higher levels of polygyny [22]. We speculate that low male reproductive skew can be expected in species with paternal care, even in the presence of strong intra-sexual competition and a male-skewed operational sex ratio.

**Fig. 1:**
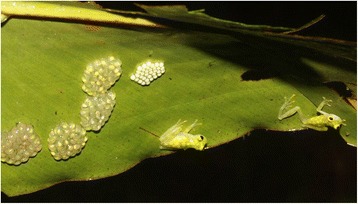
Male and female *H. valerioi* shortly after mating and egg deposition. The female (right) is leaving while the male (left) stays and guards his clutches. The egg clutch on the very right was just deposited, the other five clutches are from previous mating events

We tested this hypothesis in *Hyalinobatrachium valerioi*, a greenish-yellow glass frog (Centrolenidae) with a snout-urostyle length (SUL) of about 2 cm, which occurs in tropical rainforests from Central Costa Rica to the Pacific coast of Ecuador [23, 24]. As in most other glass frogs, members of this species are active at night and are almost exclusively observed during the rainy season when they engage in breeding activity along tropical lowland streams [25]. Males of *H. valerioi* call from elevated positions in the riverine vegetation to attract females and to announce their territories to conspecific males [26]. Once in amplexus, the female deposits a clutch of on average 29 eggs (this study) on the underside of a leaf overhanging running water. The male fertilizes the eggs and then guards the clutch while the female leaves the breeding site and abandons her offspring soon after mating [27, 28]. Successful males continue advertising for females throughout the night while they simultaneously guard up to seven clutches at various stages of development ([27]; Fig. 1). Egg clutches typically suffer from high predation [27]. After about two weeks the larvae hatch and drop into the stream below to complete their development until metamorphosis. Almost nothing is known about female mating frequencies or the genetic mating system in this species due to the inconspicuous behaviour of the females.

We performed parentage analyses of adults and larvae in a *H. valerioi* population in order to gain insights into individual mating frequencies, possible mating preferences, and reproductive skew in males and females. We expected the mating system to be at least polygynous (if not polygamous) because males guard several clutches simultaneously. For the same reason, we assumed a higher mating frequency for successful males than for females despite paternal care. And as male reproductive success is probably limited by their access to females, we expected strong intra-sexual competition among *H. valerioi* males. We chose to combine observational field data and molecular parentage analyses for our study to overcome the logistic constraints on gaining unbiased estimates of mating and reproductive success from field observations alone. Molecular parentage analyses are a powerful tool for reconstructing genealogies within populations and to elucidate individual mating patterns and sexual selection [29].

## Results

During the study period of 101 days (Aug-Nov 2012) we monitored and sampled 142 individual *H. valerioi* (93 males, 47 females, 2 sex undefined) and 374 of their larvae from 193 clutches (two larvae extracted from 181 clutches, one larva from 12 clutches). For males, we obtained 2419 focal observations in total, 1350 at night and 1069 during the day; for females we obtained 95 observations in total, 64 and 31 at night and day, respectively. Individual males were observed on average 21 times and up to 99 times (median = 21, iqr = 4–41) in the course of the study period, while most females were encountered only once (median = 1, iqr = 1–2, max = 6). The genotype of one male (m138) was not included as a candidate father for the pedigree reconstruction due to low PCR amplification success. However, since no parental genotypes were simulated by COLONY, we assumed zero mating and reproductive success for this male. The MMMeans estimator predicted a total number of 93 males at the study site, which equals the total number of males that we sampled. Since females were only recorded during mating activity, we could not estimate female sampling coverage due to the low number of recaptures.

### Parentage reconstruction

For 124 clutches (240 larvae) both assigned parents were known from the study area, and for 69 clutches (134 larvae) a known male was assigned together with a simulated female. In total, 30 female genotypes were simulated by the parentage analysis. The genotypes of the unsexed individuals were included in both the putative mothers as well as in the putative fathers for the COLONY analysis. However, both individuals were never assigned as matching parental genotypes to any of the larvae. Thus these individuals were not included in any of the further analyses. For 190 (98.45 %) out of 193 clutches the father that was observed closest to the clutch was also assigned as the genetic father by COLONY. One single clutch was attended by a different male than the one assigned in the parentage analysis. No attending male was observed at two clutches, but paternity of both clutches could be assigned to two sampled males via the parentage analysis. Most clutches (*N* = 150, 77.72 %) were found after the female had already left the breeding site, thus no predictions about maternity could be made in these cases. For the remaining 43 clutches (22.28 %) where a female was observed in amplexus with a male or close to a recent clutch, the genetic data confirmed the field observations.

### The genetic mating system and opportunity for sexual selection

**Fig. 2:**
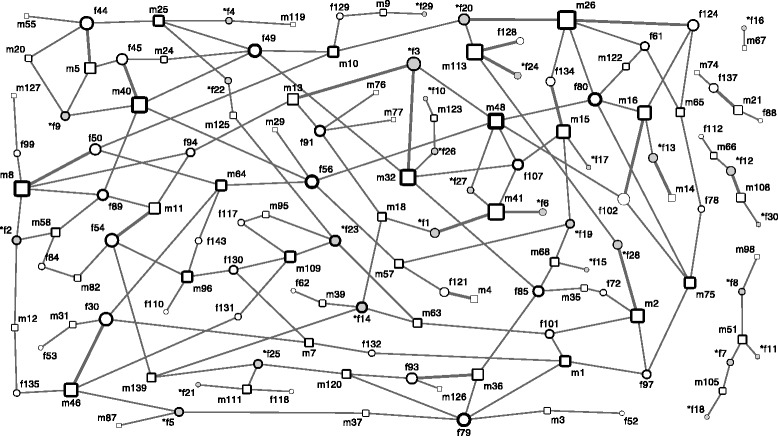
Mating network. Males (m) and females (f) are displayed as squares and circles, respectively. Simulated female genotypes (*f) are shaded in grey. The symbol size represents the number of clutches per individual (1–7), the width of the symbol outline represents the number of mating partners per individual (1–6), and the width of the edges represents the number of clutches per parent pair (1–2). Distances and locations of nodes do not correspond to the actual spatial arrangement of individuals

**Fig. 3:**
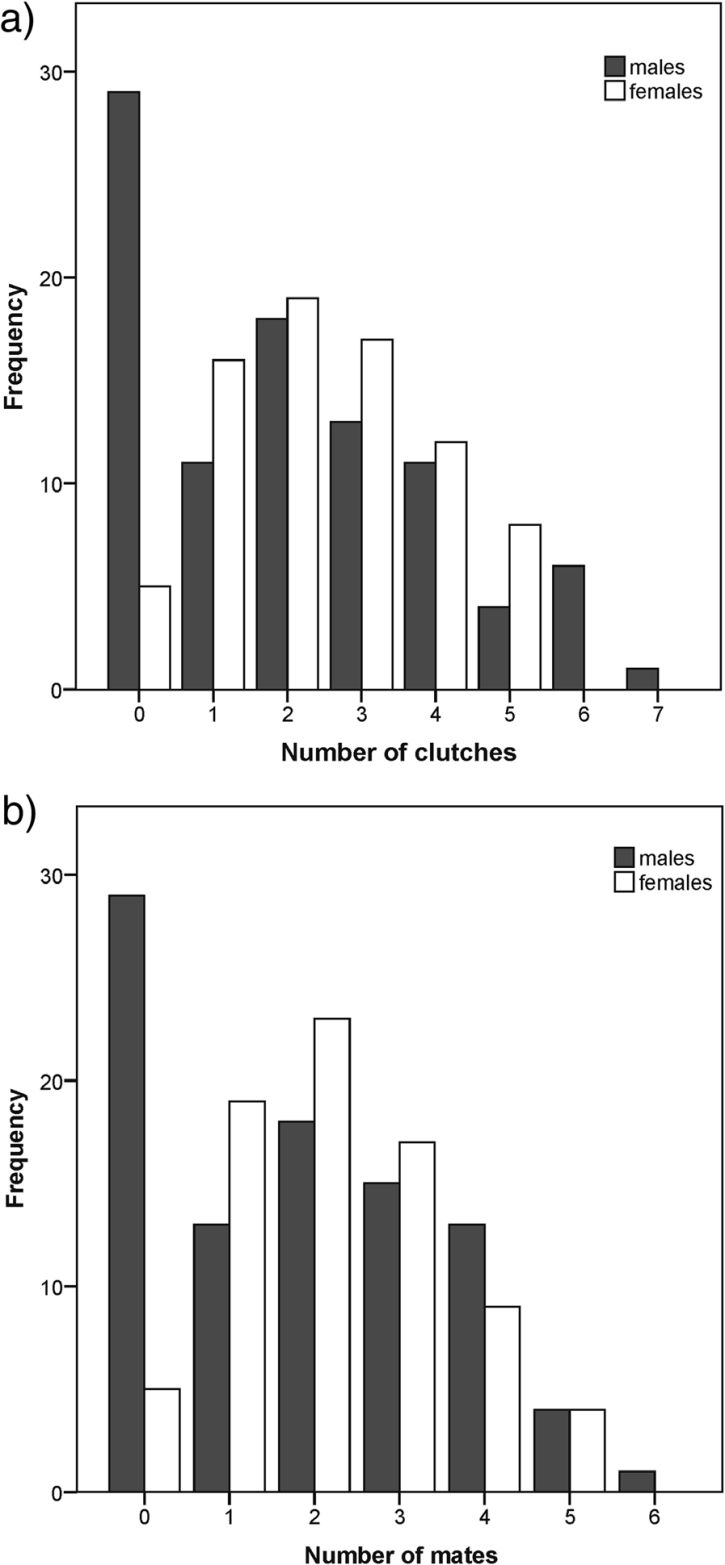
Results of the parentage analysis. (**a**) Distribution of the number of clutches and (**b**) number of mating partners per male (grey bars) and female (white bars) *H. valerioi*

**Fig. 4:**
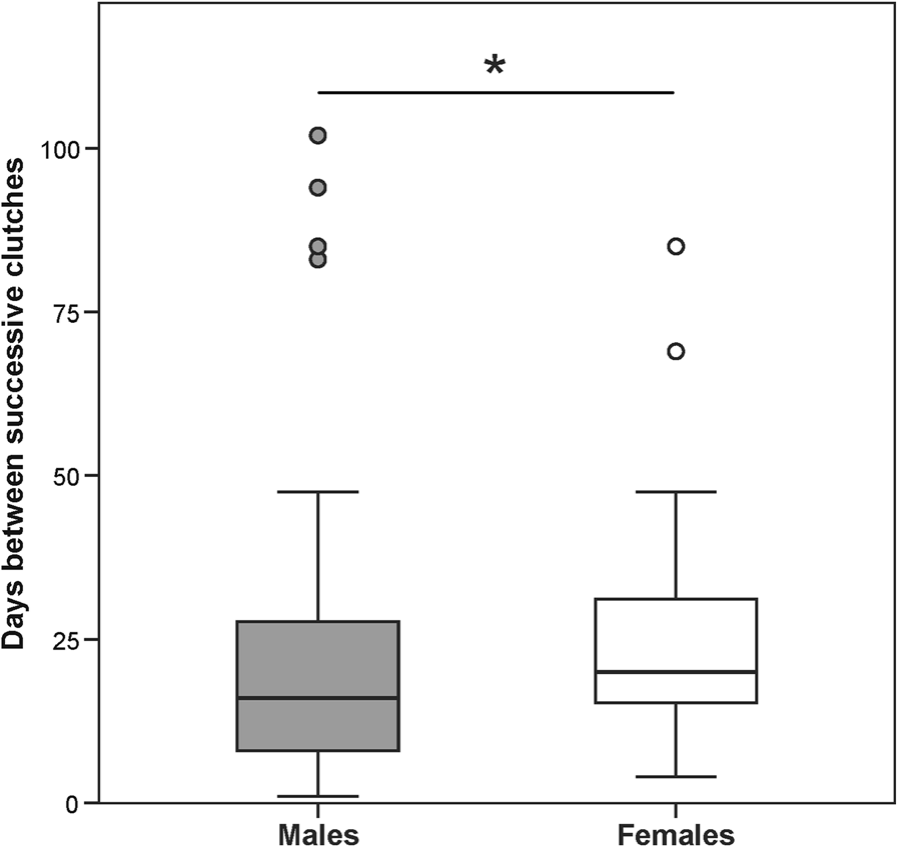
Mating frequencies. Boxplots showing distributions of mating frequencies (i.e. days between successive clutches) in males and females. Circles indicate outlier values

The mating network revealed one large cluster of connected individuals, and four small units of seven, five, four and two individuals, respectively (Fig. 2). Twenty-one pairs produced two clutches, all other 151 pairs only one clutch. Sixty-four (68.82 %) of the 93 males, and 72 (93.51 %) of the 77 females (47 sampled plus 30 simulated females) sired/produced at least one clutch (Fig. 3). This corresponds to a sex ratio in the actual reproducers of about 0.9 males per female. The mating frequency was significantly higher in males, who sired a clutch about every two weeks (median = 16 days; iqr = 8–27), than in females, who laid a clutch approximately every three weeks (median = 20 days; iqr = 14.75–31.08; Mann–Whitney *U-*test, N_m/f_ = 53/56, *U* = 1117.5, *P* = 0.026, Fig. 4).

There were significant differences in the number of clutches between males and females, while differences in the number of mates were only marginally significant (Mann–Whitney *U-*test, N_m/f_ = 93/77, clutches: *U* = 2919, *P* = 0.035; mates: *U* = 3000, *P* = 0.064). However, these differences were only due to a larger number of non-reproducers in males, as no significant differences were found for successfully reproducing males and females (Mann–Whitney *U-*test, N_m/f_ = 64/72, clutches: *U* = 2081.5, *P* = 0.321, mates: *U* = 2000.5, *P* = 0.173, Fig. 3).

Accordingly, the standardized variances of mate acquisition and clutch production were higher in males than in females (*ΔI*_*clutches*_ = 0.54, *ΔI*_*mates*_ = 0.45, Table 1), but this difference vanished when only considering successful reproducers (*ΔI*_*clutches*_ = 0.04, *ΔI*_*mates*_ = −0.02, Table 1). Accordingly, the Bateman gradients of actual reproducers did not differ significantly between the sexes (N_m/f_ = 64/72, *F*_*1,132*_ = 0.094, *P* = 0.760, Fig. 4) and showed a strong positive association between mate number and clutch production. The distribution of the number of clutches or mates per male differed significantly from a Poisson distribution when considering all individuals (Kolmogorov–Smirnov tests of goodness-of-fit: clutches: *P* = 0.003, mates: *P* = 0.024), but not for successful reproducers (Kolmogorov–Smirnov tests of goodness-of-fit: clutches: *P* = 0.998, mates: *P* = 0.928), indicating that females haphazardly mate with calling males.

**Table 1:** Opportunity for sexual selection. Standardized variances in mating and reproductive success for male and female *H. valerioi*, calculated for all individuals and successful reproducers

	All individuals			Successful reproducers		
	♂	♀		♂	♀	
Number of clutches	*I* = 0.86	*I* = 0.32	*ΔI*_clutches_ = 0.54	*I* = 0.28	*I* = 0.23	*ΔI*_clutches_ = 0.04
Number of mates	*I* = 0.78	*I* = 0.33	*ΔI*_mates_ = 0.45	*I* = 0.22	*I* = 0.24	*ΔI*_mates_ = −0.02

### Mating and reproductive success

During our daily night-time surveys we recorded 0–34 (mean ± SD = 17.76 ± 7.79) males present along the transect per night. During individual nights on average only 4.84 % of present males were found in amplexus, while the remaining 95.16 % were encountered alone. Of those males present at the reproductive site 67.82 % were also calling. The parameters ‘observation period’ (i.e. timespan between first and last day encountered), ‘#nights recorded’, and ‘#nights calling’ were all significantly correlated with each other (all rho > 0.819, all *P* < 0.001), but neither of the parameters correlated significantly with body size (all rho < 0.164, all *P* > 0.128). All three temporal parameters showed a high variation among males (‘observation period’: mean ± SD = 51.04 ± 35.97; ‘#nights recorded’: mean ± SD = 14.52 ± 12.28; ‘#nights calling: mean ± SD = 10.53 ± 9.26), while the variation in body size among males was rather low (SUL: mean ± SD = 20.74 ± 1.00). We decided to only include ‘#nights calling’ as a surrogate for all temporal parameters, because we considered this variable to have the highest biological relevance with regard to the potential impact on mating success in males. We thus performed a stepwise linear regression to test whether the parameters ‘SUL’ and/or ‘#nights calling’ were significant predictors for the number of clutches or mates per male. The parameter ‘#nights calling’ but not ‘SUL’ proved to be a significant predictor for the number of clutches (#nights calling: *t* = 5.298, *P* < 0.001; SUL: *t* = 0.329, *P* = 0.743; Model fit: R^2^ = 0.248, *F*_1,85_ = 28.071, *P* < 0.001) or mating partners per male (#nights calling: *t* = 5.003, *P* < 0.001; SUL: *t* = 0.652, *P* = 0.516; Model fit: R^2^ = 0.228, *F*_1,85_ = 25.033, *P* < 0.001). The same effect was found when the parameter ‘#nights calling’ was replaced by ‘observation period’ or ‘#nights present’.

For females, body size was not a significant predictor for the total number of clutches, the average number of eggs per clutch, or their rate of clutch production (clutches: *t* = −0.434, *F*_1,39_ = 0.188, *P* = 0.667; eggs/clutch: *t* = 0.318, *F*_1,34_ = 0.101, *P* = 0.753; mating frequency: *t* = 1.423, *F*_1,29_ = 2.024, *P* = 0.166).

### Patterns of relatedness

Among all 7084 possible male–female dyads, we observed 138 full-sibling pairs, 1216 half-sibling pairs, and 5646 unrelated pairs, corresponding to probabilities of 1.95 % for full-sib matings, 17.17 % for half-sib matings, and 79.70 % for matings between not closely related individuals when assuming fully random mating. In our actual offspring sample we detected 0.52 % of all matings to have occurred between full-siblings, 20.73 % between half-siblings and 78.76 % between not closely related individuals. The matings we observed did not significantly differ from proportions expected under random mating (2 proportions *Z*-test; full siblings: *Z* = 1.342, *P* = 0.153, half siblings: *Z* = −1.291, *P* = 0.197, unrelated: *Z* = 0.322, *P* = 0.749). The pairwise relatedness values of females and their actual mating partners did not significantly differ from the relatedness of females to the rest of males (paired t-test, *N* = 72, *t* = −0.797, *P* = 0.426).

## Discussion

### The genetic mating system and opportunity for sexual selection

For males, the MMMeans estimator equalled the number of actually sampled individuals. This indicates that for our study population the sampling of *H. valerioi* males was complete during the observation period. Females were almost exclusively recorded when engaged in mating activities, while otherwise their detection rate, and as a result their recapture rate, was very low. As the calculation of demographic estimators depends on sufficiently high recapture rates, it was thus not possible to reliably estimate female sampling coverage. However, given our long study period, we assume that all females that were in principle able to reproduce during that time were also present at the reproductive site at least once during our sampling. Since females approach males and were never observed to be rejected by males (pers. obs. KT and AM), and since male availability did not seem to be a limiting factor (only about 5 % of the males present at night were found in courtship or already amplexus), we presume that females face no general external restrictions on their opportunity to breed. Therefore we assume that the integration of information from the parentage analysis (i.e. simulated individuals) with the field observations resulted in a reliable approximation of the actual number of females in our study population, thereby yielding an unbiased and representative assessment of the reproductive activity in 2012.

Our results show a high degree of sequential polygamy in our population, where most individuals from both sexes mated more than once and with multiple partners during one single breeding season (cf. [30]; see also [20] for similar mating patterns in another frog species). For frogs, this type of mating system is generally associated with prolonged breeding seasons [31], which are particularly common in tropical environments [32] as a result of the prevailing climatic conditions. The mating network shows that reproductive activity is evenly distributed across the whole population, and that the majority of individuals form one large reproductive cluster. We observed that almost 70 % of all males recorded at the reproductive site sired at least one clutch, which is very high, compared to estimates of male mating success in other glass frog species (*H. fleischmanni*: 10 %, *C. prosoblepon*: 9 %, both based only on behavioural observations, [33]) and other anurans (cf. [10]). For the remaining 30 % of males that were unsuccessful we presume that failure to occupy suitable calling sites led to limited access to females. As the ability to establish and defend a calling site is presumably highly energetically expensive, male territoriality likely serves as honest signal for male quality to females. We did not detect alternative reproductive tactics, such as sneaking [34] or clutch piracy [35], neither during behavioural observations, nor from genetic parentage assignments. Thus, those alternative strategies might not have evolved in *H. valerioi* at all, or at least do not play a major role in male reproductive behaviour.

In our study population, we only observed five (6 %) females without reproductive success. All of these females were observed during courtship, but without apparent mating success (i.e. no clutch that could be attributed to those females was found). All other females (94 %) could be assigned to at least one clutch. Given the highly biased operational sex ratio in our study population, we propose that, in general, all females that attempt to mate will also be able to do so. Predation soon after oviposition (i.e. before we were able to genetically sample the clutch) might have been a reason for the lack of reproductive success in these females.

While males' mating success is presumably limited by their ability to advertise and defend a calling site as well as by the number of available females at the stream, females are mainly restricted physiologically by their rate of egg production (cf. [3]). Mating frequencies were significantly higher in males than in females, but this was likely due to the physiological latency (i.e. recovery time) after ovulation in females, leading to relatively few females with short mating frequencies compared to males (Fig. 4). While several studies have found a positive relationship between the number of eggs per clutch and female body size in anurans [36–38], we did not find such an association in our study, nor were the parameters ‘eggs/clutch’ or ‘mating frequency’ related to body size. Thus we conclude that body size is not a reliable predictor for female fecundity in *H. valerioi*.

**Fig. 5:**
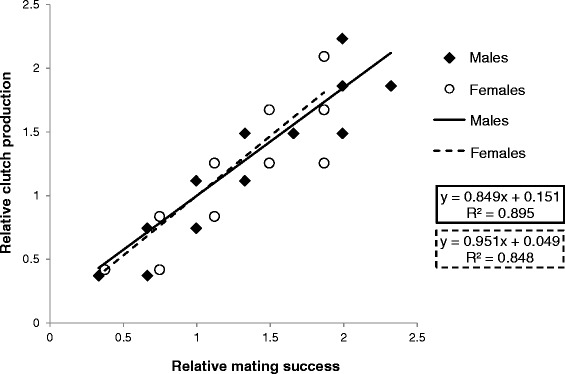
Bateman gradients for successful males and females

When looking at all individuals, individual females laid significantly more clutches on average and their number of mates was higher than for males. However, these differences were only due to a higher number of non-reproducers in males. For successful reproducers in our study population we observed a sex ratio of 0.9 males per female and the standardized variances in mate acquisition and clutch production, as well as the Bateman gradients, did not differ significantly between the sexes (Table 1, Fig. 5), indicating similar mating patterns for successful males and females. The number of mates and clutches did not significantly differ from a Poisson distribution for successful reproducers, but did differ significantly when considering all individuals. This indicates a strong intra-sexual selection among males regarding their access to females (i.e. the competition for suitable calling sites), but subsequent non-selective mating of females, resulting in an equally low opportunity for sexual selection to act on actual reproducers in our study population (cf. [2, 39]). A similar pattern has been found in a natural population of a hyper-dispersed, territorial poison frog [20].

### Being choosy or not?

Given the prolonged breeding period, high male site fidelity, and the highly male-biased operational sex ratio in the *H. valerioi* population under study, females in principle have the possibility to choose their ‘favourite’ male. However, we did not find any indication for females selecting males based on body size or relatedness. No mating preferences in *H. valerioi* females were also reported in previous studies. The clutch removal experiment by [27] showed that females do not preferentially choose males that attend a high number of clutches, although previous mating success could potentially serve as an indicator of male quality (e.g. female copying [40]). In turn, a high number of clutches might even limit the calling activity in males. Likewise, [27] did not find a correlation between male mating success and the surface area of the leaf or the height of oviposition sites.

Female preference for large males has been found in several anuran species [17]. In *H. valerioi*, bigger males might be more attractive to females as they are potentially better in defending high quality reproductive resources from other males or protecting egg clutches from approaching predators. Our findings however show that females did not preferentially mate with bigger males.

Inbreeding avoidance is known from other amphibian species (e.g. American toads, *Bufo americanus*, [41]). However, when looking at the relatedness of mating partners, our results show no preference for males of low relatedness in *H. valerioi* females, i.e. the relatedness between mating partners was as high/low as would be expected by chance. In our study population, we found a rather low risk of inbreeding (mating probability between full siblings was 2 %), and due to the lack of this selective pressure, assortative mating by relatedness might not have evolved in *H. valerioi*.

Under female choice, two general strategies might increase female reproductive success. On the one hand, if there is high variation in the quality of single males, females are expected to evolve preferences for certain male traits that serve as ‘honest signals’ for male quality [3]. On the other hand, females might also increase their reproductive success by allocating successive matings to multiple mating partners [42, 43]. Being choosy or not may also depend on the trade-off between finding the best suitable mate, and time and energy spent searching for him [44]. Moreover, benefits provided by males might decrease with increasing level of male polygyny, as is the case for parental care (cf. [21]; see also [22]). Females of *H. valerioi* have up to five mating partners during one reproductive season, one of the highest levels of polyandry reported in anurans so far [cf. 20, 45]. The fact that *H. valerioi* females rarely mate twice with the same male could be because females actively try to maximise the number of mating partners. Polyandry has repeatedly been discussed as a powerful strategy to increase fitness by spreading the risk of low quality matings, as well as to increase the genetic diversity among offspring [42, 46]. Through polyandrous mating females may also distribute clutches across many males, which might lower the risk of insufficient parental care by a single male (cf. [20, 45]; see [47] for partitioning of larvae). However, as the number of clutches and mating partners of successful males and females did not differ from a Poisson distribution, we instead interpret the observed polyandry as a result of females roaming through the breeding habitat and mating with the closest, or most prominent, calling male present at the time of ovulation (cf. [20, 48]).

### Male reproductive success

We found that only a fraction of males from the entire study population was present at the reproductive site at any individual night. The observation period, the total number of nights present at the stream, and the number of nights spent calling were significantly correlated with each other and also highly variable across males. The parameter ‘nights calling’ was taken as a proxy for all temporal parameters, because we considered this variable to be most biologically relevant concerning its potential impact on mating success in males. Indeed, the number of nights a male spent calling at the stream was a significant predictor for his mating and reproductive success. This indicates that the variance in success across males can largely be attributed to differences in time spent calling at the reproductive site. Similar results were found for the glass frog species *H. fleischmanni* and *Espadarana prosoblepon*: mating success was correlated with the number of nights a male was present at the site, but not with body size, call pitch, or call duration [33, 49]. A significant positive relationship between male mating success and the number of nights present at the reproductive site has also been found in other anuran species (e.g. *Bufo woodhousei*, [50]; *Physalaemus pustolosus*, [12]; *Bufo calamita*, [51]; *Hyla chrysoscelis*, [52]; *Phyllomedusa rohdei*, [53]; all studies based only on behavioural observation). As calling, mating, and caring can be energetically expensive, the difference in the presence of males at the reproductive site probably reflects the variance in the males’ physiological constitutions (cf. [54, 55]). Although prolonged calling activity might in principal serve as an ‘honest signal’ for male quality to females, in practice, males that call more often will at the same time automatically increase their likelihood of being chosen as a mating partner simply because they are more conspicuous than silent males. If this is the case, variation in mating success is not due to selective female choice but rather due to direct male-male competition. Nonetheless, if presence and calling at the reproductive site actually relates to male quality, females that mate with any closely calling male are more likely to mate with high-quality males without incurring costs of extended mate searching (cf. [56]).

### Low reproductive skew - a general pattern in prolonged breeders with paternal care?

In prolonged breeders, receptive females enter the breeding site successively and/or cyclically (as in the case of iteroparity), rather than simultaneously [10]. In such a highly male-skewed situation they might more easily choose their ‘best mate’ from the available males than explosive breeders. Following the same logic, but from the male perspective, this means that single ‘outstanding’ males might be able to monopolize most of the approaching females. Such a scenario is particularly likely if the ‘quality’ of a specific male – if assessable – is equally attractive to all females, and the benefits of a good male will not decrease as the number of matings increases (i.e. fixed benefits, such as ‘good genes’). However, our data did not support the hypothesis that extended breeding periods and a highly male-biased OSR lead to high reproductive skew across males. In turn, we found high levels of reproductive success in males and females. In our system, it is presumably less costly for females to be polyandrous than to achieve monandry. On the one hand, males are not present and do no call every night ([27], this study), thus a female’s previous mating partner might not always be available when she is ready to mate. When male territoriality, advertised by calling, as such is an honest signal, females who approach any nearby calling male can be sure to mate with a male of sufficient quality. On the other hand, polyandry in our study population presumably helps to further reduce the risk of repeatedly choosing a male who is genetically inferior or incompatible, or who provides insufficient parental care (cf. [57]). Fitness benefits associated with parental care distributed across several males and locations might have outbalanced any alleged benefits of single males, and thereby precluded the evolution of mate preferences in *H. valerioi*. These findings are likely to generalize to many other prolonged breeding species, particularly other vertebrates with male parental care (see [58] for a summary on parental care in vertebrates). When social and environmental conditions associated with reproduction and offspring survival are highly unpredictable, strong fitness benefits can be expected for females that spread matings across time, space, and mating partners. Such partitioning strategies might be particularly beneficial for females in species with paternal care. Typically, male parenting quality and quantity are more unpredictable than female care, as generally males have gametes more readily available than females and can more easily increase their reproductive success with additional matings than is the case for females. We propose that low male reproductive skew and high rates of polyandry might be common features in prolonged breeding species with paternal care, even in the presence of direct male-male competition, and male-biased sex ratios. Supporting evidence for this hypothesis has also been found in a recent study on mating patterns in a natural population of a hyper-dispersed, territorial frog with paternal care [20]. In this study 35.5 % of the males produced offspring that survived until adulthood, and, due to the commonly high mortality at the larval stage [59], significantly higher male mating success rates can be assumed. In this species, selection was found to act only on the males’ opportunity to breed (i.e. whether they were able to establish a territory), while females mated non-discriminatorily with the advertising males. To better understand how differential selective pressures act on mating systems and patterns of reproductive success in natural populations, comparative studies with other glass frog species that have less intense (e.g. *H. fleischmanni*, [60]) or absent (e.g. *Cochranella pulverata*, [25]) parental care are needed.

### Methodological considerations

For our study we combined observational field data with molecular parentage analyses to achieve maximal information on individual mating activities. Our results show that based on field observations alone paternity could be assigned for more than 99 % of all clutches, while for one out of 191 cases the genetic assignments did not match the field observations. This single case was due to a territory takeover after the clutch has been produced. Females were present at only 21 % of the clutches, and their maternity was confirmed in all cases by the genetic analyses. This emphasizes the advantage of molecular tools when investigating reproductive activity in species where individuals are highly cryptic, mobile and/or do not remain close to their offspring.

## Conclusions

In the present study we monitored and genetically sampled a natural population of *H. valerioi* over one breeding season and used microsatellite markers and parentage analysis to characterize the genetic mating system. Our results suggest that females are not choosy, and mate indiscriminately with available males. The variance in male mating success could largely be attributed to the variation in the time that individual males spent at the reproductive site, which in turn could reflect differences in their physiological constitutions. We found low reproductive skew in female and male *H. valerioi* and high levels of polyandry, which were amongst the highest levels of polyandry reported in anurans so far. Our findings support the hypothesis that dilutable male benefits - such as parental care - can favour female polyandry and maintain low levels of reproductive skew within a population. We show that such low levels of skew can occur even in the presence of direct male-male competition and a highly male-biased operational sex ratio. We hypothesize that low male reproductive skew is a general characteristic in prolonged breeders with paternal care.

## Methods

### Study site

The *H. valerioi* population under study is located in tropical lowland rainforest along the ‘Quebrada Negra’, a small lowland stream close to the tropical research station ‘La Gamba’, situated on the Pacific side of Costa Rica (N 8°42’61”, W 83°12’97”). Detailed characteristics of the drainage system were published by [61]. The area receives on average 5923 mm of rainfall annually with a pronounced wet season between May and November. The highest precipitation occurs from August to November, and the mean annual temperature at the site is 28.2° [62]. The study area is situated within a secondary forest located at the edge of the ‘Piedras Blancas National Park’ at 70 m above sea level. In a preliminary survey, *H. valerioi* was only detected along a 425 m section of the stream, which then was chosen as the transect for monitoring and sampling adult frogs and clutches. The stream has its source in the hilly slopes at the margins of primary forest. After passing several small waterfalls, the stream then (still very narrow at that point) flows through a small patch of primary forest in a flat area upstream of the transect. The streambed then widens to 3–5 m along the transect where *H. valerioi* is found. The stream enters open agricultural grassland downstream of the transect, habitat that is not suitable for *H. valerioi*. During our study we verified at irregular intervals that no *H. valerioi* were present for at least 150 m both up- and downstream of the transect.

### Monitoring and DNA sampling

We performed fieldwork from 15 August to 23 November 2012, covering the period with the highest breeding activity of *H. valerioi* [27]. We divided the transect into intervals of 5 m and used flagging tape as distance markers. We performed two surveys a day throughout the whole study period, one during the day (between 8 a.m. and 1 p.m.) and one at night (between 6 p.m. and 3 a.m.), with the exception of five days when high water levels due to heavy rainfall impeded field work. For each survey we slowly walked back and forth along the transect. At night we approached every calling male frog and scanned the vegetation for clutches and further frogs. We determined the exact location of frogs and clutches to the nearest meter. During the day we revisited already known clutches and calling sites to check for the presence of frogs and to sample clutches. We used a ladder to reach frogs and clutches up to a maximum height of 5 m above the streambed and bank respectively, depending on the characteristics of ground and vegetation. This dense sampling regime allowed us to gather reliable daily presence-absence data for males. The cryptic female behaviour and the accordingly much lower encounter rate only yielded presence data for females.

**Fig. 6:**
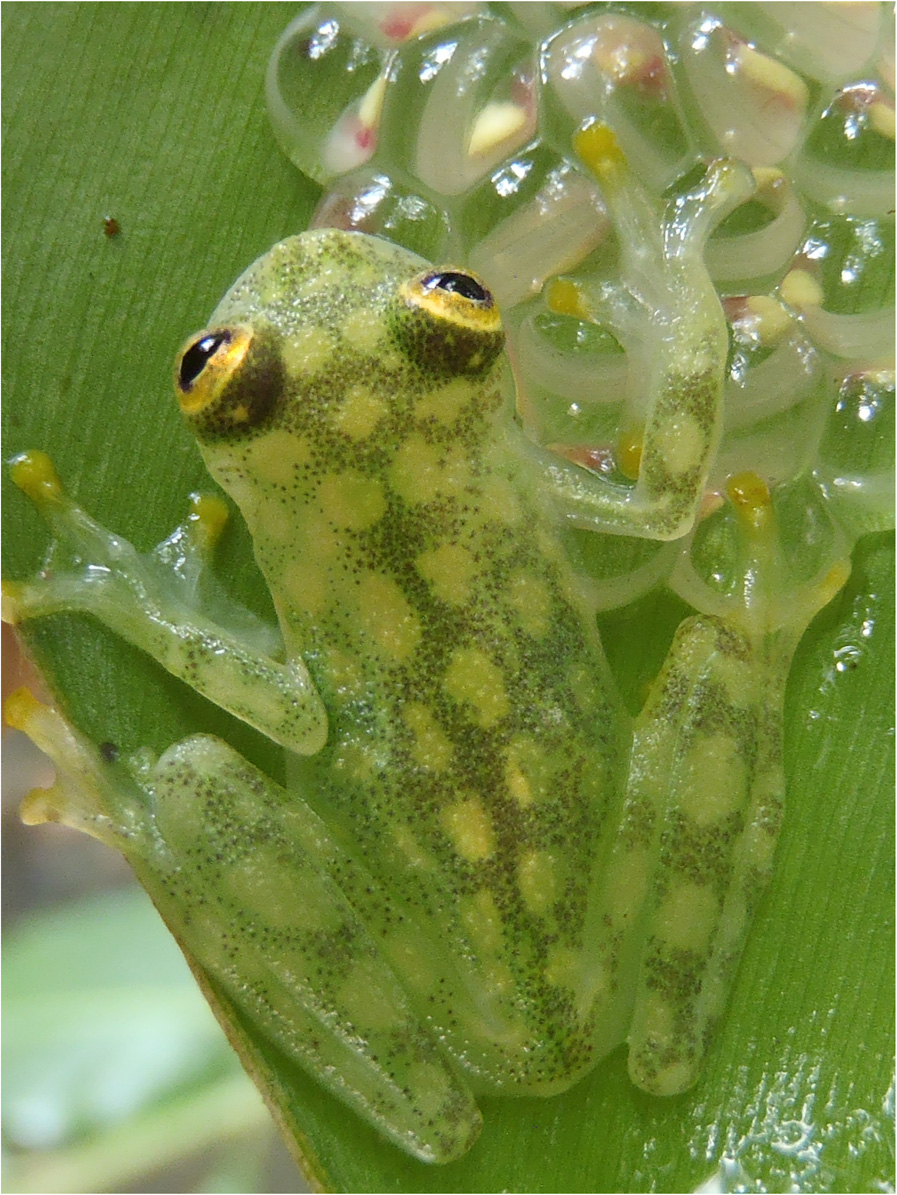
Example of a dorsal colouration pattern in an *H. valerioi* male

We took pictures of the dorsal colour pattern of all encountered frogs (Fig. 6) for individual identification. We took the first picture of an individual on scale paper for later measurements of body size (i.e. snout urostyle length, SUL) with the program ImageJ [63]. Further pictures at subsequent encounters were taken from a distance without handling the frog. We approached and checked known male positions for the male’s presence. We also took pictures of newly encountered clutches in order to later count the eggs. We collected tissue samples from all newly encountered frogs and clutches for subsequent molecular analyses. Tissue samples consisted of the pad of the third toe of both hind limbs from adult frogs and of two larvae per clutch (one for clutches with fewer than ten larvae). We released all frogs at their encounter location immediately after handling. We only sampled larvae that had at least reached Gosner stage 17 [64]. All samples were immediately preserved in 96 % ethanol. All sampling was conducted in strict accordance with Costa Rica and EU law and following the ASAB guidelines for the treatment of animals in behavioural research and teaching [65].

We determined the sampling coverage for males by calculating an asymptotic population size estimator (MMMeans, [66, 67]), based on individual capture histories using EstimateS 8.2.0. [68]. Females were almost exclusively recorded when engaged in courtship, leading to a lower detection probability and much lower recapture rate than for males, thereby precluding the reliable estimation of female sampling coverage.

### DNA extraction and microsatellite genotyping

For DNA extraction we used a Proteinase K digestion and a standard phenol-chloroform protocol [69] to isolate total genomic DNA. We amplified DNA samples at eight polymorphic microsatellite loci (*Hval04*, *Hval10*, *Hval16*, *Hval17*, *Hval20*, *Hval21*, *Hval22* and *Hval24*) using PCR primers and protocols described by [70]. After amplification, we diluted the products with water, mixed them with the internal size standard ROX350 (Applied Biosystems) and ran them on an ABI 3130xl Genetic Analyser. We identified all loci visually using PeakScanner 1.0 (Applied Biosystems) and determined final allele sizes using the binning software TANDEM v1.08 [71]. All microsatellite genotypes are given in [Additional file 1].

### Parentage analyses

We carried out parentage assignment using COLONY 2.0 [72], a program that uses a group-likelihood approach for sibship reconstruction. We used the full likelihood model with medium precision and allowed for polygamous mating in both sexes. We treated both tadpoles that had been sampled from the same clutch as ‘known maternal sibs’. Given the reproductive biology of *H. valerioi* (e.g. amplexus, male territoriality), we excluded the possibility of multiple maternity within single clutches but did not exclude the possibility of multiple paternity (e.g. through clutch piracy or delayed fertilization by sneakers). When the program could not assign a genotype from the sampled parents to a given offspring-pair, it provided a simulated parental genotype. For the following analyses of reproductive contributions we only used ‘Best (ML) Configuration’ assignments with the maximum likelihood obtained at the end of the computation (cf. COLONY user guide) [see Additional file 2].

### The genetic mating system and opportunity for sexual selection

We constructed a network graph showing all inferred matings with the program Cytoscape [73]. We tested for differences in the number of mating partners and clutches between successfully reproducing males and females with Mann–Whitney *U*-tests. We also tested for differences in the mating frequency (i.e. number of days between two successful matings) in males and females for individuals that had at least two clutches.

To identify sex-specific differences in the opportunity for selection, we calculated the standardized variance in mate acquisitions (*I*_*mates*_) and clutch production (*I*_*clutches*_) [2, 74]. This analysis was conducted for all males and females in our study population and again separately for just the successful reproducers. We also determined the Bateman gradient (the relative number of mating partners in relation to the relative number of clutches) for successfully reproducing males and females (cf. [39]).

### Male and female mating success

We investigated whether male body size and/or previous male reproductive activity were significant predictors of male reproductive success [see Additional file 3]. As the parameters ‘observation period’ (timespan between first and last day encountered), ‘#nights recorded’ (number of nights recorded), and ‘#nights calling’ (number of nights calling) were all significantly correlated with each other (see results), ‘#nights calling’ was taken as a proxy for all temporal parameters, since we considered this variable to be most biologically relevant with regard to its potential impact on mating success in males. We then performed a stepwise linear regression to test whether, for each male, body size (SUL) or ‘#nights calling’ were significantly correlated with either the number of clutches or the number of mates.

To investigate whether female body size is related to fecundity, we tested whether the parameter SUL was a significant predictor for the average number of eggs per clutch (‘eggs/clutch’), the total number of clutches per female (‘clutches’), or their mating frequency using stepwise linear regression [see Additional file 4].

We used the program KINGROUP v2 [75] to determine the pairwise relatedness coefficients *r* [76] for all male–female pairs. For the simulated individuals, we used the genotype with the highest probability as calculated by COLONY. We used the implemented simulation function to obtain reference intervals (first to third quartile) for expected pairwise relatedness values for 100 full siblings, 100 half siblings, and 100 unrelated individuals, which were [0.35;061],[0.14;0.35], and [−0.11;0.11], respectively. Accordingly, we defined full-siblings as all individual pairs with *r* values > 0.351, half siblings with values between 0.123 and 0.351, and unrelated individuals with values below 0.123. To test whether mating is random with respect to relatedness, we calculated expected probabilities for full and half siblings to mate in our study population under the assumption of random mating, and compared them with the observed numbers of matings between full and half siblings. Furthermore, we tested whether pairwise relatedness values of females and their mating partners (‘*r*_mates’) differed from their relatedness to all other males (‘*r*_nonmates’) using a paired *t*-test.

All statistical tests were performed in IBM SPSS Statistics 22.0.0. Normality of variables was tested with the Kolmogorov–Smirnov test. Medium values, interquartile ranges (iqr), and non-parametric tests were applied in those cases where normality was rejected.

## Availability of supporting data

The datasets supporting the results in this article are included in the additional files.

## Additional files

Additional file 1:
**Microsatellite genotypes of all adults and tadpoles.** (TXT 39 kb) Additional file 2:
**Summary table of clutches.** (PDF 363 kb) Additional file 3:
**Summary table of males.** (PDF 232 kb) Additional file 4:
**Summary table of females.** (PDF 294 kb)
