# Supplementary material for: Low reproductive skew despite high male-biased operational sex ratio in a glass frog with paternal care
Source: BMC Evol Biol. 2015 Sep 3;15:181. doi: 10.1186/s12862-015-0469-z (PMC4558732; doi:10.1186/s12862-015-0469-z)
Supplement: Additional file 2: — Summary table of clutches. (PDF 363 kb) [file 12862_2015_469_MOESM2_ESM.pdf]

Clutches

Legend: clutch = consecutive clutch number, oviposition = date of oviposition, # total tp = total number of tadpoles, # tp sampled = number of tadpoles sampled, ID_tp sampled = ID of sampled tadpoles, ♂/♀_field = ID of male/female observed with clutch in the field, father/mother_DNA = ID of male/female that was assigned as father/mother in the parentage analysis, match ♂/♀ = match of field observations and genetic analyses, 0 = no, 1 = yes, n/a = not applicable

clutch	oviposition	# total tp	# tp sampled	ID_tp sampled	♂_field	♀_field	father_DNA	mother_DNA	match ♂	match ♀
1	2012-08-10	24	1	145	m18	0	m18	f91	1	n/a
2	2012-08-05	25	2	146,147	m12	0	m12	f135	1	n/a
3	2012-08-04	25	2	148,149	m1	0	m1	f97	1	n/a
4	2012-08-04	30	2	150,151	m24	0	m24	f45	1	n/a
5	2012-08-06	25	2	152,153	m8	0	m8	f50	1	n/a
6	2012-08-06	28	2	154,155	m12	0	m12	*f2	1	n/a
7	2012-08-10	33	2	156,157	0	0	m82	f84	n/a	n/a
8	2012-08-05	34	2	158,159	m1	0	m1	f101	1	n/a
9	2012-08-10	31	2	160,161	m25	0	m25	f44	1	n/a
10	2012-08-10	21	2	162,163	m7	0	m7	f130	1	n/a
11	2012-08-05	30	2	164,165	m35	0	m35	f85	1	n/a
12	2012-08-18	30	2	166,167	m32	0	m32	*f3	1	n/a
13	2012-08-14	25	2	168,169	m13	0	m13	f91	1	n/a
14	2012-08-13	28	2	170,171	m26	0	m26	*f20	1	n/a
15	2012-08-16	24	2	172,173	m40	0	m40	f89	1	n/a
16	2012-08-14	26	2	174,175	m46	0	m46	*f5	1	n/a
17	2012-08-15	34	2	176,177	m37	0	m37	f79	1	n/a
18	2012-08-19	4	2	178,179	m41	0	m41	*f6	1	n/a
19	2012-08-16	4	2	180,181	m51	0	m51	*f7	1	n/a
20	2012-08-18	29	2	182,183	m51	0	m51	*f8	1	n/a

21	2012-08-23	35	2	184,185	m2	0	m2	f97	1	n/a
22	2012-08-19	23	2	186,187	m40	0	m40	*f9	1	n/a
23	2012-08-14	24	2	188,189	m41	0	m41	*f6	1	n/a
24	2012-08-20	33	2	190,191	m41	0	m41	f107	1	n/a
25	2012-08-19	20	2	192,193	m31	f30	m31	f30	1	1
26	2012-08-23	30	1	194	m21	0	m21	f88	1	n/a
27	2012-08-27	31	2	195,196	m31	f53	m31	f53	1	1
28	2012-08-27	35	2	197,198	m3	f52	m3	f52	1	1
29	2012-08-26	28	2	199,200	m57	f56	m57	f56	1	1
30	2012-08-27	34	2	201,202	m32	0	m32	f85	1	n/a
31	2012-08-28	24	2	203,204	m32	f49	m32	f49	1	1
32	2012-08-30	28	2	205,206	m32	0	m32	*f3	1	n/a
33	2012-09-01	28	2	207,208	m66	0	m66	*f12	1	n/a
34	2012-08-25	20	2	209,210	m75	0	m75	f102	1	n/a
35	2012-08-29	28	2	211,212	m75	0	m75	f97	1	n/a
36	2012-08-26	32	2	213,214	m75	0	m75	f78	1	n/a
37	2012-08-27	33	2	215,216	m75	0	m75	f80	1	n/a
38	2012-09-04	41	2	217,218	m14	0	m14	*f13	1	n/a
39	2012-09-10	34	2	219,220	m1	f79	m1	f79	1	1
40	2012-09-11	28	2	221,222	m7	f30	m7	f30	1	1
41	2012-09-06	32	2	223,224	m92	0	m63	*f14	0	n/a
42	2012-09-13	28	2	225,226	m36	f85	m36	f85	1	1
43	2012-09-15	25	2	227,228	m36	f93	m36	f93	1	1
44	2012-09-06	26	2	229,230	m15	0	m15	f134	1	n/a
45	2012-09-15	27	2	231,232	m76	f91	m76	f91	1	1
46	2012-09-14	29	2	233,234	m82	0	m82	f54	1	n/a
47	2012-09-18	35	2	235,236	m63	0	m63	*f23	1	n/a
48	2012-09-15	35	2	237,238	m98	0	m98	*f8	1	n/a
49	2012-09-18	25	2	239,240	m8	f94	m8	f94	1	1
50	2012-09-19	35	2	241,242	m65	0	m65	f61	1	n/a
51	2012-09-19	32	2	243,244	m48	0	m48	*f3	1	n/a
52	2012-09-19	39	2	245,246	m55	f44	m55	f44	1	1
53	2012-09-20	22	2	247,248	m40	f49	m40	f49	1	1

54	2012-09-20	28	2	249,250	m67	0	m67	*f16	1	n/a
55	2012-09-20	32	2	251,252	m68	0	m68	*f15	1	n/a
56	2012-09-21	34	2	253,254	m16	0	m16	*f13	1	n/a
57	2012-09-20	15	1	255	m105	0	m105	*f18	1	n/a
58	2012-09-14	35	2	256,257	m105	0	m105	*f7	1	n/a
59	2012-09-22	28	2	258,259	m108	0	m108	*f12	1	n/a
60	2012-09-22	34	2	260,261	m8	f99	m8	f99	1	1
61	2012-09-23	26	2	262,263	m16	f102	m16	f102	1	1
62	2012-09-24	39	2	264,265	m18	0	m18	*f14	1	n/a
63	2012-09-25	34	2	266,267	m96	0	m96	f143	1	n/a
64	2012-09-25	38	2	268,269	m65	f78	m65	f78	1	1
65	2012-09-25	27	2	270,271	m109	0	m109	f130	1	n/a
66	2012-09-11	33	1	272	m64	0	m64	f56	1	n/a
67	2012-09-26	33	2	273,274	m16	0	m16	f80	1	n/a
68	2012-09-28	31	2	275,276	m4	0	m4	f121	1	n/a
69	2012-09-28	35	2	277,278	m68	0	m68	f85	1	n/a
70	2012-09-28	33	2	280,281	m96	f110	m96	f110	1	1
71	2012-09-28	29	2	282,283	m46	0	m46	f30	1	n/a
72	2012-09-29	32	2	284,285	m40	0	m40	f45	1	n/a
73	2012-09-29	26	2	286,287	m66	f112	m66	f112	1	1
74	2012-10-01	39	2	288,289	m65	0	m65	f124	1	n/a
75	2012-10-03	33	2	292,293	m68	0	m68	*f19	1	n/a
76	2012-10-03	31	2	294,295	m10	0	m10	*f20	1	n/a
77	2012-10-04	27	2	296,297	m111	0	m111	*f21	1	n/a
78	2012-10-03	39	2	289,299	m20	0	m20	f44	1	n/a
79	2012-10-05	22	2	300,301	m13	0	m13	f94	1	n/a
80	2012-10-02	54	2	302,303	m14	0	m14	*f13	1	n/a
81	2012-10-04	33	2	304,305	m122	0	m122	f61	1	n/a
82	2012-10-04	26	2	306,307	m25	0	m25	*f22	1	n/a
83	2012-10-06	25	2	308,309	m25	0	m25	*f4	1	n/a
84	2012-10-04	34	2	310,311	m125	0	m125	*f23	1	n/a
85	2012-10-07	23	2	312,313	m113	0	m113	*f20	1	n/a
86	2012-10-08	27	2	314,315	m64	f50	m64	f50	1	1

87	2012-10-08	27	2	316,317	m109	f117	m109	f117	1	1
88	2012-10-08	28	2	318,319	m13	0	m13	*f3	1	n/a
89	2012-10-11	28	2	320,321	m111	0	m111	f118	1	n/a
90	2012-10-08	28	2	322,323	m125	0	m125	*f22	1	n/a
91	2012-10-13	32	2	324,325	m109	0	m109	f131	1	n/a
92	2012-10-10	36	2	326,327	m122	0	m122	f80	1	n/a
93	2012-10-10	26	2	328,329	m113	0	m113	*f24	1	n/a
94	2012-10-12	33	2	330,331	m127	0	m127	f99	1	n/a
95	2012-10-13	23	2	332,333	m111	0	m111	*f25	1	n/a
96	2012-10-14	34	2	334,335	m109	0	m109	*f23	1	n/a
97	2012-10-14	34	2	336,337	m36	f93	m36	f93	1	1
98	2012-10-11	27	2	338,339	m11	f89	m11	f89	1	1
99	2012-10-11	21	2	340,341	m36	f79	m36	f79	1	1
100	2012-10-11	31	2	342,343	m4	f121	m4	f121	1	1
101	2012-10-13	23	2	344,345	m10	f49	m10	f49	1	1
102	2012-10-14	34	2	346,347	m26	f124	m26	f124	1	1
103	2012-10-14	28	2	348,349	m11	0	m11	f54	1	n/a
104	2012-10-16	25	2	350,351	m32	0	m32	*f26	1	n/a
105	2012-10-16	23	2	352,353	m11	0	m11	f94	1	n/a
106	2012-10-17	23	2	354,355	m15	0	m15	*f19	1	n/a
107	2012-10-10	34	2	356,357	m2	0	m2	f101	1	n/a
108	2012-10-16	33	2	358,359	m74	0	m74	f137	1	n/a
109	2012-10-19	43	2	360,361	m5	0	m5	f44	1	n/a
110	2012-10-17	38	2	362,363	m46	f30	m46	f30	1	1
111	2012-10-18	30	2	364,365	m58	0	m58	f84	1	n/a
112	2012-10-22	17	2	366,367	m58	0	m58	*f2	1	n/a
113	2012-10-25	27	2	368,369	m95	0	m95	f117	1	n/a
114	2012-10-22	26	2	370,371	m15	0	m15	*f17	1	n/a
115	2012-10-22	30	2	372,373	m123	0	m123	*f26	1	n/a
116	2012-10-25	29	2	374,375	m13	0	m13	*f3	1	n/a
117	2012-10-26	27	2	376,377	m48	0	m48	f56	1	n/a
118	2012-10-27	26	1	378	m63	0	m63	f101	1	n/a
119	2012-10-22	36	2	379,381	m10	f129	m10	f129	1	1

120	2012-10-25	31	2	382,383	m10	0	m10	f50	1	n/a
121	2012-10-23	38	2	384,385	m95	0	m95	*f23	1	n/a
122	2012-10-25	30	2	386,387	m20	0	m20	*f9	1	n/a
123	2012-10-25	33	2	388,389	m41	0	m41	*f27	1	n/a
124	2012-10-26	29	2	390,391	m96	f130	m96	f130	1	1
125	2012-10-26	37	2	392,393	m21	f137	m21	f137	1	1
126	2012-10-25	30	2	394,395	m15	0	m15	f107	1	n/a
127	2012-10-19	26	2	396,397	m113	0	m113	f128	1	n/a
128	2012-10-26	27	2	399,400	m2	0	m2	*f28	1	n/a
129	2012-10-26	25	2	401,402	m1	f132	m1	f132	1	1
130	2012-10-27	27	2	403,404	m113	0	m113	*f24	1	n/a
131	2012-10-27	38	2	405,406	m16	0	m16	f124	1	n/a
132	2012-10-27	22	2	407,409	m77	0	m77	f91	1	n/a
133	2012-10-28	26	1	408	m24	f49	m24	f49	1	1
134	2012-10-29	32	1	410	m46	f135	m46	f135	1	1
135	2012-11-02	35	2	411,424	0	0	m18	*f1	n/a	n/a
136	2012-10-30	23	2	412,423	m123	0	m123	*f10	1	n/a
137	2012-10-29	40	2	413,414	m5	0	m5	f44	1	n/a
138	2012-10-31	40	2	415,416	m2	0	m2	f72	1	n/a
139	2012-10-29	25	2	417,418	m15	f134	m15	f134	1	1
140	2012-10-29	28	2	419,420	m120	0	m120	f79	1	n/a
141	2012-10-29	32	2	421,422	m57	0	m57	f121	1	n/a
142	2012-10-29	21	2	425,426	m16	0	m16	f102	1	n/a
143	2012-10-29	19	2	427,428	m96	0	m96	f54	1	n/a
144	2012-10-28	30	2	429,430	m119	0	m119	*f4	1	n/a
145	2012-10-24	1	1	431	m39	0	m39	*f14	1	n/a
146	2012-10-28	31	2	432,433	m39	0	m39	f62	1	n/a
147	2012-10-31	26	2	434,435	m126	f93	m126	f93	1	1
148	2012-10-31	31	2	436,437	m40	0	m40	f45	1	n/a
149	2012-11-01	29	2	438,439	m87	0	m87	*f5	1	n/a
150	2012-11-01	36	2	440,441	m64	f30	m64	f30	1	1
151	2012-10-30	14	2	442,443	m21	f137	m21	f137	1	1
152	2012-10-30	30	1	444	m48	0	m48	f80	1	n/a

153	2012-11-02	26	2	445,446	m8	f89	m8	f89	1	1
154	2012-11-04	35	2	447,448	m41	0	m41	*f1	1	n/a
155	2012-11-04	29	2	449,450	m40	f56	m40	f56	1	1
156	2012-11-06	33	2	452,453	m64	f143	m64	f143	1	1
157	2012-11-07	34	2	454,455	m48	0	m48	f107	1	n/a
158	2012-11-07	25	2	456,457	m120	0	m120	*f25	1	n/a
159	2012-11-06	31	2	458,459	m8	0	m8	*f2	1	n/a
160	2012-11-05	42	2	460,461	m108	0	m108	*f30	1	n/a
161	2012-11-08	37	1	462	m108	0	m108	*f12	1	n/a
162	2012-11-08	31	2	463,464	m8	f50	m8	f50	1	1
163	2012-11-05	34	2	465,466	m9	0	m9	f129	1	n/a
164	2012-11-09	38	2	467,468	m26	0	m26	f61	1	n/a
165	2012-11-08	35	2	469,470	m57	0	m57	*f19	1	n/a
166	2012-11-12	35	1	471	m48	0	m48	*f27	1	n/a
167	2012-11-13	27	2	472,473	m48	0	m48	f102	1	n/a
168	2012-11-10	39	2	474,475	m26	f124	m26	f124	1	1
169	2012-11-13	24	2	476,477	m7	f132	m7	f132	1	1
170	2012-11-10	33	2	478,479	m41	0	m41	*f1	1	n/a
171	2012-11-12	25	2	480,481	m5	0	m5	f45	1	n/a
172	2012-10-15	32	2	482,483	m139	0	m139	*f14	1	n/a
173	2012-10-10	24	2	484,485	m139	0	m139	*f25	1	n/a
174	2012-11-04	4	1	486	m139	0	m139	f54	1	n/a
175	2012-11-11	29	2	487,488	m51	0	m51	*f11	1	n/a
176	2012-11-14	20	2	489,490	m11	0	m11	f54	1	n/a
177	2012-11-15	24	2	491,492	m113	0	m113	f128	1	n/a
178	2012-11-15	38	2	493,494	m35	0	m35	f72	1	n/a
179	2012-11-14	35	2	495,496	m26	0	m26	*f20	1	n/a
180	2012-11-13	27	2	497,498	m26	0	m26	f80	1	n/a
181	2012-11-13	31	2	499,500	m25	0	m25	f49	1	n/a
182	2012-11-17	17	2	524,525	m26	0	m26	f134	1	n/a
183	2012-11-17	29	2	502,503	m46	0	m46	f131	1	n/a
184	2012-11-17	33	2	504,505	m9	0	m9	*f29	1	n/a
185	2012-11-17	25	2	506,507	m37	0	m37	*f5	1	n/a

186	2012-11-18	33	2	508,509	m3	0	m3	f79	1	n/a
187	2012-11-17	30	2	510,511	m5	0	m5	*f9	1	n/a
188	2012-11-15	32	2	512,513	m2	0	m2	*f28	1	n/a
189	2012-11-19	22	2	514,515	m113	0	m113	*f28	1	n/a
190	2012-11-20	35	2	516,517	m32	0	m32	f107	1	n/a
191	2012-11-20	32	2	518,519	m29	0	m29	f56	1	n/a
192	2012-11-20	27	2	520,521	m58	0	m58	f89	1	n/a
193	2012-11-21	29	2	522,523	m120	0	m120	f93	1	n/a
