# Supplementary material for: Low reproductive skew despite high male-biased operational sex ratio in a glass frog with paternal care
Source: BMC Evol Biol. 2015 Sep 3;15:181. doi: 10.1186/s12862-015-0469-z (PMC4558732; doi:10.1186/s12862-015-0469-z)
Supplement: Additional file 3: — Summary table of males. (PDF 232 kb) [file 12862_2015_469_MOESM3_ESM.pdf]

Males

Legend: ID = individual identity, # clutches = number of clutches, # mates = number of mating partners, SUL = snout-urostyle-length, observation period = timespan (days) between first and last day encountered, # nights recorded = number of nights recorded, # nights calling = number of nights calling, mating rate = average number of days between consecutive matings.

ID	# clutches	# mates	SUL	observation period	#nights present	#nights calling	mating frequency
m1	4	4	20.91	83	32	28	27.67
m10	4	4	21.14	85	32	20	7.33
m100	0	0	22.83	1	1	1	n/a
m105	2	2	21.33	1	1	0	6.00
m106	0	0	21.78	1	1	1	n/a
m108	3	2	n/a	49	10	6	23.50
m109	4	4	20.70	27	15	7	6.33
m11	4	3	19.90	100	27	22	11.33
m111	3	3	19.83	21	11	6	4.50
m113	6	4	18.25	55	7	5	8.60
m114	0	0	20.28	6	5	5	n/a
m115	0	0	21.85	1	1	1	n/a
m116	0	0	19.95	14	6	5	n/a
m119	1	1	19.33	43	10	9	n/a
m12	2	2	22.17	1	1	1	1.00
m120	3	3	n/a	44	20	14	11.50
m122	2	2	21.05	6	2	2	6.00
m123	2	2	20.49	17	8	8	8.00
m125	2	2	20.47	2	2	1	4.00
m126	1	1	n/a	29	15	10	n/a
m127	1	1	19.58	1	0	0	n/a
m13	4	3	20.46	82	26	20	24.00
m133	0	0	20.03	7	5	5	n/a

m138	0	0	20.15	2	2	1	n/a
m139	3	3	20.14	23	2	2	12.50
m14	2	1	21.24	62	20	10	28.00
m140	0	0	19.53	1	1	1	n/a
m144	0	0	n/a	9	4	4	n/a
m15	5	4	21.10	81	21	17	13.25
m16	5	4	19.72	84	23	16	9.50
m17	0	0	19.86	56	7	4	n/a
m18	3	3	21.87	56	22	14	42.00
m19	0	0	21.30	75	6	4	n/a
m2	5	4	22.23	100	23	17	21.00
m20	2	2	21.28	85	35	20	22.00
m21	3	2	22.70	90	17	12	34.00
m22	0	0	19.60	16	3	2	n/a
m24	2	2	21.58	92	21	15	85.00
m25	4	4	20.27	97	11	8	31.67
m26	7	5	20.71	99	28	18	16.00
m27	0	0	20.54	71	17	14	n/a
m28	0	0	20.95	47	18	11	n/a
m29	1	1	20.76	97	24	22	n/a
m3	2	2	22.56	101	38	31	83.00
m31	2	2	21.13	12	11	3	8.00
m32	6	5	20.84	97	23	12	18.80
m34	0	0	20.73	36	3	3	n/a
m35	2	2	21.61	97	17	14	102.00
m36	4	3	19.57	90	49	40	10.33
m37	2	2	20.93	97	31	28	94.00
m38	0	0	21.25	1	1	1	n/a
m39	2	2	20.25	80	2	2	4.00
m4	2	1	20.53	100	49	31	13.00
m40	6	5	22.42	77	33	25	16.00
m41	6	4	21.01	87	24	12	17.60
m42	0	0	22.29	1	1	1	n/a

m43	0	0	20.81	84	28	17	n/a
m46	5	4	20.73	90	35	23	23.75
m47	0	0	21.40	70	2	1	n/a
m48	6	6	20.89	81	30	21	11.00
m5	4	3	19.46	99	18	16	9.67
m51	3	3	22.94	87	3	3	43.50
m55	1	1	19.61	77	39	32	n/a
m57	3	3	20.78	85	18	12	37.00
m58	3	3	21.90	88	10	8	16.50
m6	0	0	20.51	54	11	9	n/a
m60	0	0	19.32	35	14	5	n/a
m63	3	3	20.27	67	17	14	25.50
m64	4	4	21.35	78	27	18	18.67
m65	3	3	20.79	43	11	9	6.00
m66	2	2	20.56	34	15	8	28.00
m67	1	1	21.15	22	2	2	n/a
m68	3	3	19.76	35	18	12	6.50
m69	0	0	20.80	1	1	1	n/a
m7	3	3	22.01	99	26	19	47.50
m70	0	0	18.87	1	1	1	n/a
m71	0	0	19.86	1	1	1	n/a
m73	0	0	21.61	26	6	2	n/a
m74	1	1	19.55	59	21	18	n/a
m75	4	4	18.79	6	3	2	1.33
m76	1	1	20.44	31	14	7	n/a
m77	1	1	21.43	56	9	6	n/a
m8	6	5	21.30	92	39	33	18.80
m81	0	0	20.06	1	1	0	n/a
m82	2	2	21.25	54	7	7	35.00
m86	0	0	17.97	57	5	5	n/a
m87	1	1	19.61	67	25	21	n/a
m9	2	2	21.00	101	21	20	12.00
m90	0	0	22.12	57	14	12	n/a

m92	0	0	21.16	7	1	1	n/a
m95	2	2	21.36	42	4	4	2.00
m96	4	4	n/a	64	27	16	11.33
m98	1	1	n/a	1	1	1	n/a
