# Supplementary material for: Low reproductive skew despite high male-biased operational sex ratio in a glass frog with paternal care
Source: BMC Evol Biol. 2015 Sep 3;15:181. doi: 10.1186/s12862-015-0469-z (PMC4558732; doi:10.1186/s12862-015-0469-z)
Supplement: Additional file 4: — Summary table of females. (PDF 294 kb) [file 12862_2015_469_MOESM4_ESM.pdf]

# Females

**Legend:** ID = individual identity, # clutches = number of clutches, # mates = number of mating partners, SUL = snout-urostyle-length, eggs/clutch = average number of eggs per clutch, mating frequency = average number of days between consecutive matings, *r* _mates = average relatedness to mating partners, *r* _nonmates = average relatedness to non-chosen males.

| ID | # clutches | # mates | SUL | eggs/clutch | mating frequency | *r* _mates | *r* _nonmates |
|---|---|---|---|---|---|---|---|
| f30 | 5 | 4 | 21.88 | 30.20 | 18.50 | -0.07 | -0.05 |
| f44 | 5 | 4 | 21.04 | 38.40 | 20.00 | -0.09 | -0.03 |
| f45 | 4 | 3 | n/a | 29.50 | 33.33 | -0.11 | -0.01 |
| f49 | 5 | 5 | 21.04 | 25.20 | 19.25 | -0.06 | 0.04 |
| f50 | 4 | 3 | n/a | 28.50 | 31.33 | -0.02 | 0.06 |
| f52 | 1 | 1 | n/a | 35.00 | n/a | -0.18 | -0.07 |
| f53 | 1 | 1 | 21.26 | 31.00 | n/a | 0.02 | 0.09 |
| f54 | 5 | 4 | 21.05 | 20.00 | 15.80 | 0.01 | 0.03 |
| f56 | 5 | 5 | 24.47 | 29.80 | 21.50 | -0.08 | 0.04 |
| f59 | 0 | 0 | 21.99 | n/a | n/a | n/a | n/a |
| f61 | 3 | 3 | 22.36 | 35.33 | 25.50 | 0.00 | -0.02 |
| f62 | 1 | 1 | 22.15 | 31.00 | n/a | -0.03 | -0.06 |
| f72 | 2 | 2 | 22.31 | 39.00 | 15.00 | -0.15 | -0.10 |
| f78 | 2 | 2 | n/a | 35.00 | 30.00 | -0.06 | -0.01 |
| f79 | 5 | 5 | 21.33 | 30.00 | 23.75 | 0.02 | 0.05 |
| f80 | 5 | 5 | 23.81 | 31.80 | 19.50 | 0.00 | -0.10 |
| f83 | 0 | 0 | 20.77 | n/a | n/a | n/a | n/a |
| f84 | 2 | 2 | 22.58 | 31.50 | 69.00 | 0.15 | -0.06 |
| f85 | 4 | 4 | 21.63 | 31.75 | 18.00 | -0.05 | -0.03 |
| f88 | 1 | 1 | 21.43 | 30.00 | n/a | -0.16 | -0.08 |
| f89 | 4 | 4 | 21.94 | 26.00 | 32.00 | -0.04 | -0.06 |
| f91 | 4 | 4 | 20.22 | 24.50 | 26.00 | 0.15 | 0.00 |
| f93 | 4 | 3 | 22.04 | 28.50 | 22.33 | 0.13 | -0.06 |

| | | | | | | | | |
|---|---|---|---|---|---|---|---|---|
| f94 | 3 | 3 | 20.83 | 23.33 | 14.00 | 0.03 | -0.05 |
| f97 | 3 | 3 | 19.22 | 29.33 | 12.50 | 0.02 | 0.01 |
| f99 | 2 | 2 | 22.38 | 33.50 | 20.00 | -0.19 | -0.15 |
| f101 | 3 | 3 | 21.36 | 31.33 | 41.50 | -0.05 | -0.01 |
| f102 | 4 | 3 | 22.22 | 23.50 | 26.67 | -0.07 | 0.03 |
| f107 | 4 | 4 | 21.83 | 33.00 | 30.67 | -0.01 | 0.01 |
| f110 | 1 | 1 | 21.16 | 33.00 | n/a | -0.27 | -0.01 |
| f112 | 1 | 1 | n/a | 26.00 | n/a | -0.09 | -0.04 |
| f117 | 2 | 2 | 21.47 | 27.00 | 17.00 | 0.15 | 0.00 |
| f118 | 1 | 1 | 23.80 | 28.00 | n/a | -0.11 | 0.01 |
| f121 | 3 | 2 | 20.76 | 31.33 | 15.50 | -0.02 | 0.06 |
| f124 | 4 | 3 | 21.54 | 37.50 | 13.33 | -0.11 | 0.01 |
| f128 | 2 | 1 | 21.97 | 25.00 | 27.00 | 0.14 | -0.02 |
| f129 | 2 | 2 | 21.01 | 35.00 | 14.00 | 0.03 | -0.05 |
| f130 | 3 | 3 | 22.17 | 25.67 | 38.50 | 0.01 | 0.02 |
| f131 | 2 | 2 | 22.31 | 30.50 | 35.00 | -0.15 | 0.04 |
| f132 | 2 | 2 | 22.37 | 24.50 | 18.00 | 0.06 | -0.02 |
| f134 | 3 | 2 | 23.37 | 22.67 | 36.00 | 0.02 | -0.01 |
| f135 | 2 | 2 | 21.74 | 28.50 | 85.00 | -0.05 | -0.01 |
| f136 | 0 | 0 | 22.76 | n/a | n/a | n/a | n/a |
| f137 | 3 | 2 | n/a | 28.00 | 7.00 | 0.00 | 0.08 |
| f141 | 0 | 0 | 22.53 | n/a | n/a | n/a | n/a |
| f142 | 0 | 0 | 22.09 | n/a | n/a | n/a | n/a |
| f143 | 2 | 2 | 23.60 | 33.50 | 42.00 | -0.16 | 0.02 |
| *f01 | 3 | 2 | n/a | 34.33 | 4.00 | n/a | n/a |
| *f02 | 3 | 3 | n/a | 25.33 | 46.00 | n/a | n/a |
| *f03 | 5 | 3 | n/a | 29.40 | 17.00 | n/a | n/a |
| *f04 | 2 | 2 | n/a | 27.50 | 22.00 | n/a | n/a |
| *f05 | 3 | 3 | n/a | 26.67 | 47.50 | n/a | n/a |
| *f06 | 2 | 1 | n/a | 14.00 | 5.00 | n/a | n/a |
| *f07 | 2 | 2 | n/a | 19.50 | 29.00 | n/a | n/a |
| *f08 | 2 | 2 | n/a | 32.00 | 28.00 | n/a | n/a |
| *f09 | 3 | 3 | n/a | 27.67 | 45.00 | n/a | n/a |

| | | | | | | | |
|---|---|---|---|---|---|---|---|
| *f10 | 1 | 1 | n/a | 23.00 | n/a | n/a | n/a |
| *f11 | 1 | 1 | n/a | 29.00 | n/a | n/a | n/a |
| *f12 | 3 | 2 | n/a | 31.00 | 34.00 | n/a | n/a |
| *f13 | 3 | 2 | n/a | 43.00 | 14.00 | n/a | n/a |
| *f14 | 4 | 4 | n/a | 26.00 | 16.00 | n/a | n/a |
| *f15 | 1 | 1 | n/a | 32.00 | n/a | n/a | n/a |
| *f16 | 1 | 1 | n/a | 28.00 | n/a | n/a | n/a |
| *f17 | 1 | 1 | n/a | 26.00 | n/a | n/a | n/a |
| *f18 | 1 | 1 | n/a | 15.00 | n/a | n/a | n/a |
| *f19 | 3 | 3 | n/a | 30.33 | 16.33 | n/a | n/a |
| *f20 | 4 | 3 | n/a | 29.25 | 31.00 | n/a | n/a |
| *f21 | 1 | 1 | n/a | 27.00 | n/a | n/a | n/a |
| *f22 | 2 | 2 | n/a | 27.00 | 4.00 | n/a | n/a |
| *f23 | 4 | 4 | n/a | 35.25 | 11.67 | n/a | n/a |
| *f24 | 2 | 1 | n/a | 26.50 | 17.00 | n/a | n/a |
| *f25 | 3 | 3 | n/a | 24.00 | 14.00 | n/a | n/a |
| *f26 | 2 | 2 | n/a | 27.50 | 6.00 | n/a | n/a |
| *f27 | 2 | 2 | n/a | 34.00 | 18.00 | n/a | n/a |
| *f28 | 3 | 2 | n/a | 27.00 | 12.00 | n/a | n/a |
| *f29 | 1 | 1 | n/a | 33.00 | n/a | n/a | n/a |
| *f30 | 1 | 1 | n/a | 42.00 | n/a | n/a | n/a |
